# Mechanistic insights into cAMP-mediated presynaptic potentiation at hippocampal mossy fiber synapses

**DOI:** 10.3389/fncel.2023.1237589

**Published:** 2023-07-13

**Authors:** Ryota Fukaya, Rinako Miyano, Himawari Hirai, Takeshi Sakaba

**Affiliations:** ^1^Institute for Biology/Genetics, Freie Universität Berlin, Berlin, Germany; ^2^Graduate School of Brain Science, Doshisha University, Kyoto, Japan

**Keywords:** hippocampal mossy fiber synapses, cAMP-mediated potentiation, presynaptic plasticity, synaptic vesicle release, readily-releasable pool

## Abstract

Presynaptic plasticity is an activity-dependent change in the neurotransmitter release and plays a key role in dynamic modulation of synaptic strength. Particularly, presynaptic potentiation mediated by cyclic adenosine monophosphate (cAMP) is widely seen across the animals and thought to contribute to learning and memory. Hippocampal mossy fiber-CA3 pyramidal cell synapses have been used as a model because of robust presynaptic potentiation in short- and long-term forms. Moreover, direct presynaptic recordings from large mossy fiber terminals allow one to dissect the potentiation mechanisms. Recently, super-resolution microscopy and flash-and-freeze electron microscopy have revealed the localizations of release site molecules and synaptic vesicles during the potentiation at a nanoscale, identifying the molecular mechanisms of the potentiation. Incorporating these growing knowledges, we try to present plausible mechanisms underlying the cAMP-mediated presynaptic potentiation.

## Introduction

Neurotransmitters are released from presynaptic terminals and bound to postsynaptic receptors, carrying neuronal signals from one cell to another. When an action potential (AP) arrives at the presynaptic terminal, voltage-gated Ca^2+^ channels are activated and mediate Ca^2+^ influx that initiates exocytosis of synaptic vesicles (SVs) filled with neurotransmitters. This process takes place in the nanoscopic domains called active zones (AZs), defined and structured by a set of molecules organizing the release (Südhof, 2012; Sakamoto et al., 2018; Walter et al., 2018). The amounts of SV release, together with postsynaptic factors such as the number and the density of receptors, sets weighting of the synaptic transmission (Atwood and Karunanithi, 2002). The SV release is dynamically regulated by neuronal activities, shaping presynaptic plasticity, which brings a plastic nature to neural circuit computation (Jackman and Regehr, 2017; Monday et al., 2018). For transmitter release, the processes of SV docking/priming, Ca^2+^ sensing and fusion are crucial. However, the molecular changes underlying presynaptic plasticity largely remains elusive.

Glutamatergic synapses between mammalian hippocampal mossy fibers and CA3 pyramidal cells (MF-CA3 synapses) show cAMP-mediated presynaptic potentiation (Weisskopf et al., 1994; Nicoll and Schmitz, 2005), one of the well documented types of plasticity across the animals thought to underlie learning and memory (Kandel, 2001; Heisenberg, 2003; Castillo, 2012). The cAMP-mediated presynaptic potentiation at the MF-CA3 synapses is induced by tetanic stimulation of presynaptic neurons, dentate gyrus granule cells (GCs), via protein kinase A (PKA) activation (Zalutsky and Nicoll, 1990; Weisskopf et al., 1994; Huang and Kandel, 1996). One CA3 pyramidal cell receives a single synaptic contact from one GC (Acsády et al., 1998; Delvendahl et al., 2013), and a large size of hippocampal mossy fiber boutons (hMFBs) allows for direct presynaptic patch-clamp recordings (Geiger and Jonas, 2000; Hallermann et al., 2003) and live imaging (Regehr et al., 1994; Kamiya et al., 2002; Midorikawa and Sakaba, 2017). Due to the simple induction protocols and the unique anatomical features, the MF-CA3 synapse has been used as a model for presynaptic potentiation.

It is generally considered that SVs are released from the SV population ready to be released by stimulation (readily-releasable pool; RRP) (Zucker and Regehr, 2002; Rizzoli and Betz, 2005; Kaeser and Regehr, 2017). The neurotransmitter release is often described as a function of the number of SVs within the RRP (N_RRP_) and release probabilities of SVs in the RRP (P_r_). It is unclear whether N_RRP_ is the same as N (the number of release sites) defined from the quantal hypothesis of SV release (del Castillo and Katz, 1954; Kaeser and Regehr, 2017; Pulido and Marty, 2017; Sakaba, 2018). Here, we use the term N_RRP_ as the number of SVs released by presynaptic depolarization, as described below. Electrophysiological analyses are used to examine if either an increase in N_RRP_ or P_r_ is responsible for cAMP-mediated potentiation at MF-CA3 synapses. However, the results are equivocal: Some studies rather support an increase of N_RRP_ (Vandael et al., 2020), while others support an increase in P_r_ (Weisskopf et al., 1994; Fukaya et al., 2023), or both (Reid et al., 2004). Recent developments of super-resolution microscopy and flash-and-freeze electron microscopy have allowed linking the physiological outcomes with localizations of AZ molecules and SVs during the potentiation (Imig et al., 2020; Vandael et al., 2020; Fukaya et al., 2023). We here incorporate these recent findings and try to present a coherent view of mechanistic changes underlying cAMP-mediated presynaptic potentiation.

## SV docking/priming underlying regulation of P_r_

**FIGURE 1:**
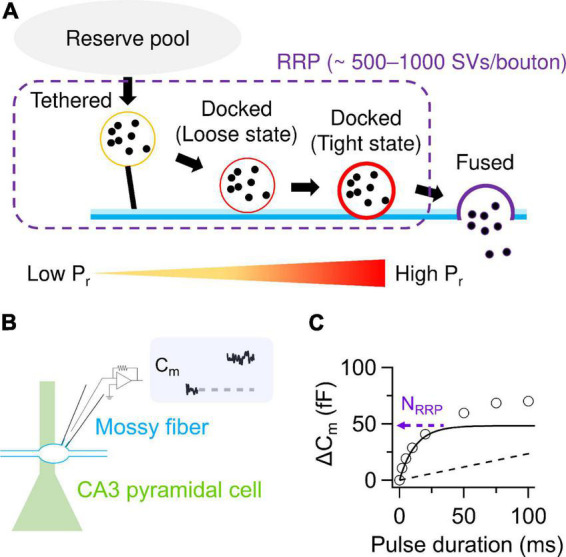
SV release parameters at hMFBs. **(A)** Schematic illustration of SV dynamics in the RRP at hMFBs. As a tethered SV becomes docked loosely then tightly, P_r_ of the SV is thought to increase with progression of molecular priming. It has been proposed that tethered and loosely- and tightly-docked SVs are located ∼50 nm, ∼8–10 nm, and within 2–4 nm from the presynaptic plasma membrane, respectively (Neher and Brose, 2018). **(B)** Scheme of direct presynaptic patch-clamp recordings at MF-CA3 synapses. Membrane capacitance (C_m_) recorded under voltage clamp condition allows for quantification of SV release, because an increase in C_m_ linearly correlates with the number of exocytosed SVs estimated from the EPSCs (∼0.1 fF/SV). **(C)** C_m_ increases under the basal condition are plotted against the durations of depolarization (circles). The release can be dissected into exponential (solid) and linear components (dashed), representing release from the RRP and the following replenishment of the RRP, respectively. The amplitude of the exponential component provides an estimate of N_RRP_. Note that a faster time course of the exponential component represents higher P_r_. In panels **(B,C)**, the trace and the plotted data are presented in Fukaya et al. (2023).

Recent conceptual advances offer molecular mechanisms of P_r_. SVs undergo tethering and docking/priming at the release sites (Sakaba, 2018). It has been considered that the docked SVs are classified into loosely- and tightly-docked SVs, detected as a difference in distances from presynaptic plasma membrane, and that P_r_ increases in the course of SVs getting docked loosely and then tightly to the release sites (Neher and Brose, 2018; Figure 1A). The tightening might advance molecular priming and increase fusion competence, presumably corresponding to progression of zippering of SNARE complex (Neher and Brose, 2018). The idea of loose and tight docking states may be comparable to such concepts as “slow and fast releasing pools” (Sakaba, 2006; Ritzau-Jost et al., 2014), “primed and super-primed SVs” (Lee et al., 2012; Taschenberger et al., 2016) and “replacement and docking sites” (Miki et al., 2016). However, correspondence among these terms needs further characterization.

The fusion process requires Ca^2+^ binding to the sensor proteins of the SVs (Eggermann et al., 2011; Jackman and Regehr, 2017). In addition to the Ca^2+^ sensing properties, the coupling distance between Ca^2+^ channels and docked SVs is also crucial for P_r_ (“positional priming”) (Wadel et al., 2007; Neher and Sakaba, 2008). Tighter coupling is mechanistically realized by shorter physical distances between Ca^2+^ channels and SVs and larger Ca^2+^ influx in the AZs. It follows that fusion competence and Ca^2+^ channel-SV release coupling synergistically determine P_r_.

## SV release parameters at basal MF-CA3 synapses

**FIGURE 2:**
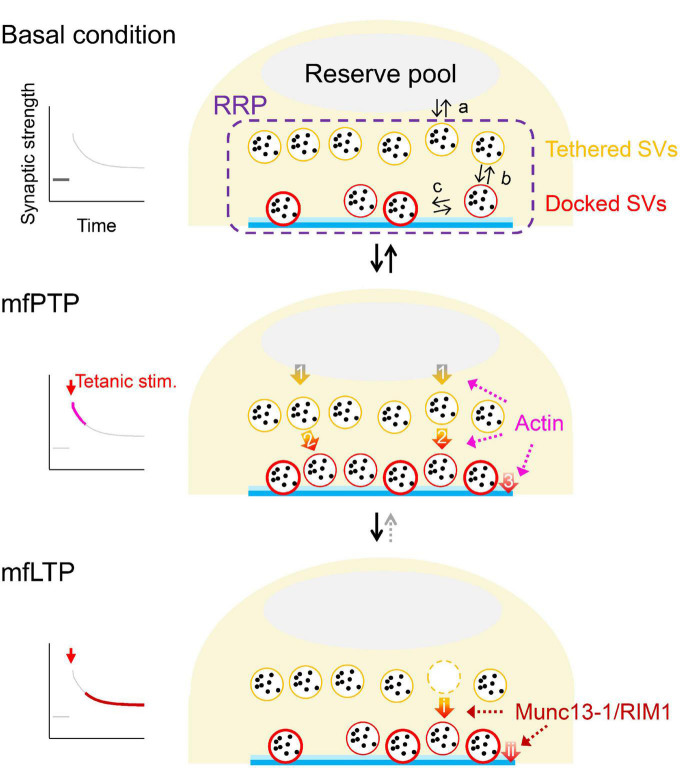
Possible mechanistic changes underlying mfPTP and mfLTP. Schematic illustrations of the RRP in the basal condition **(top)** and during mfPTP **(middle)** and mfLTP **(bottom)**. In **(top)**, equilibriums between the reserve pool and RRP (a), between undocked/tethered and docked states of the SVs in the RRP (b) and between loose and tight states of the docked SVs (c) are indicated. The mfPTP phase would return to the basal situation, if tetanic stimulation is not strong enough to permit mfLTP, where the potentiation seems more solidified by the increases of AZ proteins and less reversible. At the PTP phase, N_RRP_ and the number of docked SVs are increased. SV replenishment to the RRP (arrow 1) and/or docking actions of SVs (arrow 2) could be promoted, helping some docked SVs to become tightly docked (arrow 3). It is also possible that the docking states are directly modulated (arrow 3). Actin could be involved in these three steps potentially (see text). At the LTP phase, Munc13-1 and RIM1 are increased, increasing fusion competence of the docked SVs. These increased AZ components might stabilize the docked SVs in a tight state, by regulating docking state directly (arrow ii) and/or promoting docking of SVs that helps docked SVs to roll into a tight state (arrows i→ii). In ether scenario, N_RRP_ is unchanged, and P_r_ is increased.

Direct presynaptic patch-clamp recording in hMFBs allows one to quantify the SV release as an increase in membrane capacitance caused by SV exocytosis (Hallermann et al., 2003). Depolarization strong enough to deplete the RRP provides estimation of the N_RRP_ around 500–1,000 SVs/bouton (Hallermann et al., 2003; Miyano et al., 2019; Fukaya et al., 2023; Figures 1B, C). The RRP is depleted with a time constant of ∼10–40 ms and refilled with that of hundreds of milliseconds (Miyano et al., 2019; Fukaya et al., 2023). N_RRP_ seems larger than the number of tightly-docked SVs (∼300 SVs/bouton) and rather matches the number of SVs localized within ∼50 nm of the AZs (∼900 SVs/bouton), which includes both docked and undocked (i.e., tethered) SVs (Rollenhagen et al., 2007; Maus et al., 2020; Figures 1A, 2). Nevertheless, whether undocked/tethered SVs are included in the RRP or not is a matter of debate (Kaeser and Regehr, 2017). It is appreciated that docked SVs might be a major SV source for AP-evoked release (Imig et al., 2014, 2020; Borges-Merjane et al., 2020). One AP triggers release of ∼2–20 SVs/bouton in most cases (Jonas et al., 1993; Chamberland et al., 2014; Vyleta and Jonas, 2014; Vandael et al., 2020). This value seems equivalent to at most ∼10% of the total N_RRP_. The basal low P_r_ of the hMFBs has been explained by loose Ca^2+^ channel-docked SV coupling, characterized by relatively long physical distances in between (∼80 nm) and fast Ca^2+^ buffering in the AZs (Vyleta and Jonas, 2014).

A large N_RRP_ with low P_r_ of MF-CA3 synapses is a typical feature of high-pass filtering “tonic” synapses (Neher and Brose, 2018), showing prominent synaptic facilitation during train stimulation (Salin et al., 1996; Geiger and Jonas, 2000). P_r_ increases as the repetitive stimulation goes on via broadening of an AP waveform (Geiger and Jonas, 2000; but see Chamberland et al., 2014). Activation of presynaptic kainate (Lauri et al., 2001; Schmitz et al., 2001; but see Kwon and Castillo, 2008) or NMDA receptors (Lituma et al., 2021), Ca^2+^ release from intracellular organelles (Shimizu et al., 2008) or saturation of Ca^2+^ buffers in the AZs (Vyleta and Jonas, 2014) can also contribute to the facilitation.

## Induction protocols for cAMP-mediated potentiation in hMFBs

Tetanic stimulation has been applied by bulk electrical stimulation at the mossy fiber tract. Recent studies have used single-bouton stimulation with a presynaptic patch-clamp electrode (Vyleta and Jonas, 2014; Vyleta et al., 2016; Vandael et al., 2020, 2021) and optogenetic tools enabling tetanic stimulation by light illumination (Ben-Simon et al., 2015; Fukaya et al., 2023), successfully inducing robust potentiation. Ca^2+^ entry at hMFBs during tetanic stimulation plays a role in induction of potentiation afterward (Castillo et al., 1994; Tong et al., 1996; Breustedt et al., 2003), presumably leading to cAMP/PKA activation mediated by adenylate cyclase 1 (Huang and Kandel, 1996) and 8 (Wang et al., 2003). After tetanic stimulation is applied to GC axons, the synaptic response immediately amplifies ∼5-fold and then attenuates to the basal level in several minutes (Griffith, 1990), showing post-tetanic potentiation (PTP). When repetition and length of the train stimulation are increased to a sufficient level, PTP is followed by ∼2-fold potentiation lasting for at least tens of minutes, shaping long-term potentiation (LTP) (Zalutsky and Nicoll, 1990). Both PTP and LTP are suppressed by PKA inhibitors at MF-CA3 synapses (Weisskopf et al., 1994; Vandael et al., 2020), while, at other synapses, presynaptic PTP and LTP can be induced by different molecular pathways such as protein kinase C cascade (Alle et al., 2001; Korogod et al., 2007; Castillo, 2012). For clarification, PTP and LTP in hMFBs are herein termed mfPTP and mfLTP, respectively.

In addition to tetanic stimulation, pharmacological activation of cAMP/PKA pathway with cAMP analogues or an adenylate cyclase activator, forskolin, has also been used for potentiation (“chemical potentiation”) (Weisskopf et al., 1994; Midorikawa and Sakaba, 2017; Fukaya et al., 2021; Orlando et al., 2021). Although the chemical potentiation is robust and prevails throughout the preparation, it unlikely shares the induction pathway completely with mfPTP or mfLTP: Some presynaptic molecules, such as Rab3a and RIM1alpha, are responsible for mfLTP, not for chemical potentiation (Castillo et al., 1997, 2002), and vice versa (Shahoha et al., 2022).

## Mechanistic changes underlying mfPTP and mfLTP

Vandael et al. (2020) induced mfPTP via a cell-attached presynaptic patch electrode and analyzed postsynaptic currents recorded from the paired CA3 pyramidal cell. They concluded that mfPTP is attributed largely to an increase in N_RRP_, while a slight increase in P_r_ can also contribute. In addition, the flash-and-freeze electron microscopy revealed that the number of the docked SVs is increased within a minute after the optical tetanic stimulation. Interestingly, such changes have been also observed, when chemical potentiation is induced by 15 min forskolin application (Orlando et al., 2021).

The increased docked SVs during mfPTP might be caused by (1) increased refilling rate from the reserve pool to the RRP (arrow 1 in Figure 2) and/or (2) increased rate of SV docking (arrow 2 in Figure 2). It is also possible that (3) promoting loosely-docked SVs into tightly-docked ones (arrow 3 in Figure 2) contributes to mfPTP (Taschenberger et al., 2016). In addition to the increased forward rates, the increased number of docked SVs may be caused by a shift in the equilibrium between undocked and docked states (arrows b in Figure 2, top, Hosoi et al., 2007). At the calyx of Held, Lee et al. (2010) have proposed such a scenario. Vandael et al. (2020) have suggested that actin depolymerization reduced the steady state of the EPSC and PTP, which led them to suggest that the enhanced SV refilling to the RRP was responsible for mfPTP (arrow 1 in Figure 2). The studies at other synapses rather suggest that actin polymerization is required for docking (arrow 2 in Figure 2, Lee et al., 2012) or SV supply from replacement sites to docking sites (Miki et al., 2016). There is no direct evidence that actin is involved in transition from loose to tight states defined by Neher and Brose (2018) (arrow 3 in Figure 2).

Fukaya et al. (2021) and Midorikawa and Sakaba (2017) have reported that a short application of forskolin or cAMP analogues (<10 min, the time course similar or slightly longer than PTP) increases P_r_ with unchanged N_RRP_. This potentiation is induced mainly by tightening Ca^2+^ channel-SV release coupling via accumulation of Ca^2+^ channels near the release sites. The mechanism is different from mfPTP and longer application of forskolin, involving increases in N_RRP_ and the number of docked SVs (Vandael et al., 2020; Orlando et al., 2021). It remains to be seen if Ca^2+^ channel accumulation happens physiologically.

Fukaya et al. (2023) have introduced photo-activated cation channels to GCs to induce mfLTP by optical tetanic stimulation. After the optical LTP induction, we performed presynaptic patch-clamp recordings and membrane capacitance measurements in the photo-sensitive hMFBs. We found that, in contrast to mfPTP, P_r_ is increased by mfLTP induction, while N_RRP_ is not changed. Nevertheless, mfLTP is not caused by a change in the coupling between Ca^2+^ channels and SVs, but by an increase in fusion competence of the vesicles in the RRP. Importantly, stimulated emission deletion microscopy suggested that Munc13-1 and RIM1, which are involved in docking/priming of the SVs (Betz et al., 2001), are increased in the AZs after mfLTP induction (Fukaya et al., 2023), consistent with contributions of these proteins to mfLTP (Castillo et al., 2002; Yang and Calakos, 2011; but see Kaeser et al., 2008). These proteins are putative release site molecules and rather control the number of release sites and N_RRP_ at the synapses with high P_r_ (Sakamoto et al., 2018). How can changes of the docking/priming molecules account for an increase in P_r_, not in N_RRP_?

N_RRP_ in hMFBs is ∼20–40 SVs/AZ while the RRP likely contains both undocked and docked SVs (Maus et al., 2020), meaning that only limited number of SVs in the RRP can access to and be clamped at the release sites, a process essential for molecular priming. It is presumed that the increased priming molecules improve the accessibility and/or stabilize the SV-release site complex, consequently increasing the number of docked SVs in a tight state. The increased Munc13-1/RIM1 during mfLTP might act on (1) docking of the SVs (arrow i in Figure 2), which in turn supplies tightly-docked SVs (arrow ii in Figure 2), and/or (2) tightening of the docked SVs (arrow ii in Figure 2), which could be accompanied with compensatory enhancement of SV docking (arrow i in Figure 2). A change in the docking state may underlie presynaptic LTP at other hippocampal synapses (Jung et al., 2021). The faster replenishment of RRP is also triggered during the mfLTP phase (Fukaya et al., 2023), but the change does not seem large enough to influence N_RRP_ at rest. Rather, this acceleration could help SV supply to maintain evoked release during repetitive APs.

## Future outlook

Recent advances have started to identify molecular mechanisms of presynaptic potentiation at mossy fiber synapses. The studies mentioned above suggest involvements of actin in mfPTP and Munc13-1/RIM1 in mfLTP, but these are not exclusive, and other molecules may contribute to mfPTP and mfLTP. It should be also noted that Figure 2 is a mechanistic proposal at the current stage. More detailed electrophysiological analyses along with molecular or pharmacological perturbations and applications of dynamic release models (Pan and Zucker, 2009; Vyleta and Jonas, 2014; Miki et al., 2016; Taschenberger et al., 2016; Ritzau-Jost et al., 2018; Miyano et al., 2019; Kobbersmed et al., 2020) will help to understand the cellular/molecular mechanisms of plasticity quantitively. In consequence of mfPTP and mfLTP, the synaptic filtering properties can be modified dynamically (Monday et al., 2018), influencing the circuit computation. Identification of molecular mechanisms for the potentiation will help to elucidate a plastic nature of the circuit function.

Potentiation mechanisms via cAMP/PKA cascade seem different between tetanic stimulation and chemical induction, and are also different depending on the intensity of the inductions, as described above (mfPTP vs. mfLTP). Variations in spatiotemporal patterns of cAMP concentration and resultant enzymatic activities likely contribute, in line with the fact that genetic ablation of a PKA-anchoring protein, AKAP7, suppresses chemical potentiation, but not mfPTP and mfLTP (Jones et al., 2016). Note that another cAMP-dependent protein, Epac2, can also contribute to mfPTP and mfLTP (Fernandes et al., 2015).

During mfLTP, Munc13-1 and RIM1 are increased in the AZs, while other presynaptic molecules such as Rab3a (Castillo et al., 1997), Synaptotagmin 12 (Kaeser-Woo et al., 2013), and Tomosyn (Ben-Simon et al., 2015) can also contribute to mfLTP. However, how these proteins cooperate for the potentiation entailing AZ reorganization has not yet been resolved. It has been reported that cytoskeletal activities and protein synthesis are involved in mfLTP (Barnes et al., 2010; Monday et al., 2022). A recent study revealed that local actin synthesis at hMFBs increases the terminal volume, underlying mfLTP over an hour after the induction (Monday et al., 2022). It is interesting to see if the AZ reorganization is accompanied with this enlargement. AZ reorganization has been also observed during homeostatic presynaptic potentiation at hippocampal synapses (Glebov et al., 2017; Müller et al., 2022) and at Drosophila neuromuscular junctions (Böhme et al., 2019; Mrestani et al., 2021; Ghelani et al., 2023). This Drosophila homeostatic potentiation recruits presynaptic molecules involved in olfactory associative memory formation in flies (Turrel et al., 2022). This may warrant investigations of how AZ reorganization operates presynaptic plasticity, to elucidate molecular mechanisms underlying learning and memory in the animals.

## Author contributions

All authors wrote the manuscript and prepared the figures.
